# Stage-specific expression of Toll-like receptors in the seminiferous epithelium of mouse testis

**DOI:** 10.1007/s00418-024-02310-z

**Published:** 2024-07-31

**Authors:** Göksel Doğan, Mustafa Sandıkçı, Levent Karagenç

**Affiliations:** Faculty of Veterinary Medicine, Department of Histology-Embryology, Aydın Adnan Menderes University, 09000 Aydın, Turkey

**Keywords:** Germ cell, Mouse, Testis, Spermatogenesis, Toll-like receptors

## Abstract

Genes encoding Toll-like receptors (TLRs) are expressed by germ cells in the mouse testis. Nevertheless, the expression of TLRs by germ cells has only been demonstrated for TLR-3, TLR-9, and TLR-11. Furthermore, the expression of each TLR in relation to the stage of spermatogenesis remains uncertain. We aimed in the present study to examine the expression pattern of all TLRs in germ cells throughout the cycle of seminiferous epithelium in the adult mouse testis. Immunohistochemistry was used to evaluate the expression of TLRs. Results of the present study reveal the expression of TLRs by specific populations of germ cells. Expression of TLRs, except for TLR-7, at endosomal compartments, acrosomes, and/or residual bodies was another interesting and novel finding of the present study. We further demonstrate that the expression of TLR-1, -2, -3, -4, -5, -7, -11, -12, and -13 follows a distinct spatiotemporal pattern throughout the cycle of seminiferous epithelium. While TLR-1, -3, -5, -11, and -12 are expressed in all stages, TLR-4 is expressed only in early and middle stages of spermatogenic cycle. On the other hand, TLR-2, -7, and -13 are expressed only in early stage of spermatogenic cycle. Evidence demonstrating the expression of TLRs in a stage specific manner throughout spermatogenesis strengthen the hypothesis that the expression of various TLRs by germ cells is a developmentally regulated process. However, if TLRs play a role in the regulation of proliferation, growth, maturation, and differentiation of germ cells throughout the cycle of the seminiferous epithelium warrants further investigations.

**Supplementary Information:**

The online version contains supplementary material available at 10.1007/s00418-024-02310-z.

## Introduction

Toll-like receptors (TLRs) that recognize pathogen-associated molecular patterns (PAMPs) are the best characterized pattern recognition receptors (PRRs) (Janeway and Medzhitov 2002; Roach et al. 2005; O’Neill and Bowie 2007; Hedger 2011). Binding of various ligands to specific TLRs triggers an innate immune response (Kawai and Akira 2006; Ward 2012), a prerequisite for the killing and clearance of various pathogens (Alexopoulou et al. 2001). Although 28 types of TLRs (TLR1 to TLR28) have been identified in vertebrates (Nie et al. 2018), mammals have only 13 TLRs (TLR1 − 13) (Akira et al. 2001; Roach et al. 2005; Takeda and Akira 2005). Of these, TLR-10 is a nonfunctional pseudogene in mice (Hasan et al. 2005; Lee et al. 2014). Although TLRs are mainly expressed by immune cells such as the dendritic cells, monocytes or macrophages (Underhill 2004), they are also expressed in various nonimmune tissues and organs (Cudicini et al. 1997; Cario and Podolsky 2000; Akhtar et al. 2003; Melmed et al. 2003; Smith et al. 2003; Schaefer et al. 2004; Zhang et al. 2004; Lauw et al. 2005; Girling and Hedger 2007; Palladino et al. 2007, 2008; Nagaosa et al. 2009), including the testis tissue (Palladino et al. 2007; Bhushan et al. 2008; Wu et al. 2008;2018; Shang et al. 2011; Wang et al. 2012; Chen et al. 2014; Saeidi et al. 2014; Nejsum et al. 2019; Sun et al. 2019; Özbek et al. 2020; Öztop et al. 2020).

It has been suggested that TLRs might be involved in the regulation of various testicular functions (Hedger 2011) including spermatogenesis (Girling and Hedger 2007). Spermatogenesis, viz. development of functional spermatozoa, is a highly complex and developmentally regulated process initiated at the puberty, involving continuous and serial events of cellular proliferation and differentiation of germ cells within the seminiferous epithelium (Kimmins et al. 2004). This, what is referred to as the cycle of the seminiferous epithelium, occurs in twelve sequential stages (stages I–XII) in the mouse based on the periodic acid Schiff (PAS)–hematoxylin staining (Oakberg 1956). At stages I–VII, acrosomic granules occur and acrosome spreads over the periphery of the nucleus. At stage VIII, the acrosome moves away from the nucleus and approaches the surface of the cytoplasm. At this stage, step 16 elongated spermatids are delivered to the lumen through spermiation. While two generations of spermatids (round and elongated; steps 1–16) are present within the seminiferous epithelium at the first eight stages, only elongated spermatids (steps 9–12) are seen at stages IX–XII. At stages IX–XII, elongated spermatids are defined by their morphology and condensation of the chromatin (Oakberg 1956; Meistrich 1993; Hess and Renato de Franca 2008; Ahmed and de Rooij 2009; Meistrich and Hess 2013).

There are several studies demonstrating that TLRs are expressed by germ cells both in the mouse (Wang et al. 2012; Chen et al. 2014; Nejsum et al. 2019; Sun et al. 2019) and the rat (Bhushan et al. 2008; Özbek et al. 2020; Öztop et al. 2020). Nevertheless, in the mouse, the expression pattern of TLRs by specific populations of germ cells has only been demonstrated for TLR-3, TLR-9, and TLR-11. Furthermore, spatiotemporal expression of TLRs in relation to the cycle of the seminiferous epithelium remains largely unknown. With these in mind, we examined in the present study the expression of all functional TLRs in the adult mouse testis in sequential sections stained with specific antibodies and PAS–hematoxylin in an attempt to reveal the expression of each TLR by germ cells throughout the cycle of the seminiferous epithelium. While confirming previous observations for the expression of TLR-11 and TLR-3, the present study further reveals a distinct and stage-specific pattern of expression for all functional TLR in the mouse testis.

## Materials and methods

### Animals

Aguti F2 mice (C57BL/6× BALBc) were maintained on a 14 h light:10 h dark photoperiod (light on at 5 am) with free access to food and water. The experimental protocol was approved by the institutional Animal Ethics Committee of Adnan Menderes University, Aydin, Turkey (protocol no: 64583101/2019/121). Testis tissue samples of males were used to examine the expression of TLRs. Since all TLR antibodies used in the current study have been recently tested to show expression patterns in mouse lung tissues prepared in 4% paraformaldehyde/phosphate-buffered saline (PBS) fixation, testis tissues were fixed in 4% paraformaldehyde/PBS (pH 7.4) at 4 °C for 24 h instead of Bouin’s fixation. All tissue samples were dehydrated through a graded series of ethanol and were embedded in Paraplast X-TRA (Leica, Germany). Periodic acid Schiff (PAS) and immunohistochemical staining were performed on sequential sections taken at 100 µm intervals.

### PAS-hematoxylin staining and immunohistochemistry

To determine the stage of the seminiferous epithelium, periodic acid Schiff (PAS) staining was used as described previously (Ahmed and de Rooij 2009). Thin (5 μm) tissue sections were deparaffinized and incubated in 1% periodic acid for 30 min at room temperature. Sections were washed in running water for 10 min and were incubated for 45 min in Schiff’s reagent. Following one more wash step in running water, sections were rinsed in distilled water and were counterstained with Mayer’s hematoxylin for 3 min. Images were captured using an Olympus BX51 microscope equipped with an Olympus DP70 camera and DP controller software (Olympus, Ver. 3.1.1.267).

Immunohistochemistry was used to detect germ cells expressing TLR-1–13 (except for TLR-10) as described previously (Doğan et al. 2024a; 2024b). Histostain Plus Broad-Spectrum kit (Invitrogen) was used for the detection of TLRs. Working conditions of all TLR antibodies were previously optimized in our previous study in which specific protein bands of all TLRs were also demonstrated in the mouse testis tissue by western blotting method (Doğan et al. 2024a). Anti-TLR-1 (B-23, Sc-130896, Santa Cruz Biotechnology, 1/50), anti-TLR 2 (NB100-56720, Novus, 1/50), anti-TLR-3 (NB100-56571, Novus, 1/50), anti-TLR-4 (NB100- 56,566, Novus, 1/50), anti-TLR-5 (H-127, Sc-10742, Santa Cruz Biotechnology, 1/50), anti-TLR-6 (NBP1-54,336, Novus, 1/50), anti-TLR-7 (NB100-56682, Novus, 1/50), anti-TLR-8 (NBP2- 24,917, Novus, 1/50), anti-TLR-9 (NBP2-24,729, Novus, 1/50), anti-TLR-11 (NBP1-77,204, Novus, 1/50), anti-TLR-12 (NBP2-24,833, Novus, 1/50), and anti-TLR-13 (NBP2-24,539, Novus, 1/50) were used as primary antibodies. TLRs were detected using 3,3′-diaminobenzidine tetrahydrochloride solution (DAB; 3 mg/mL in Tris–HCl, pH 7.6, with 3% H_2_O_2_). Sections treated in an identical manner except for the use of TBS (pH 7.6) instead of a primary antibody were used as negative controls. Mayer’s hematoxylin was used for counter-staining. Images were captured using an Olympus BX51 microscope equipped with an Olympus DP70 camera and DP controller software (Olympus, Ver. 3.1.1.267).

## Results

In an attempt to demonstrate the expression pattern of all functional TLRs throughout the cycle of the seminiferous epithelium, we performed PAS staining for staging of the seminiferous epithelium (Supplementary Fig. 1) along with immunohistochemistry on sequential sections (Supplementary Figs. 2–4). Microscopic evaluation of the sections at low magnification (40×) reveals the expression of TLR-1,-2, -3 and -4 (Supplementary Fig. 2); TLR-5 and -7 (Supplementary Fig. 3); andTLR-11, -12, and -13 (Supplementary Fig. 4) by germ cells at specific stages in the cycle of the seminiferous epithelium. No immune positivity was detected in any of the negative control sections used for each antibody (Supplementary Fig. 5).

**Fig. 1:**
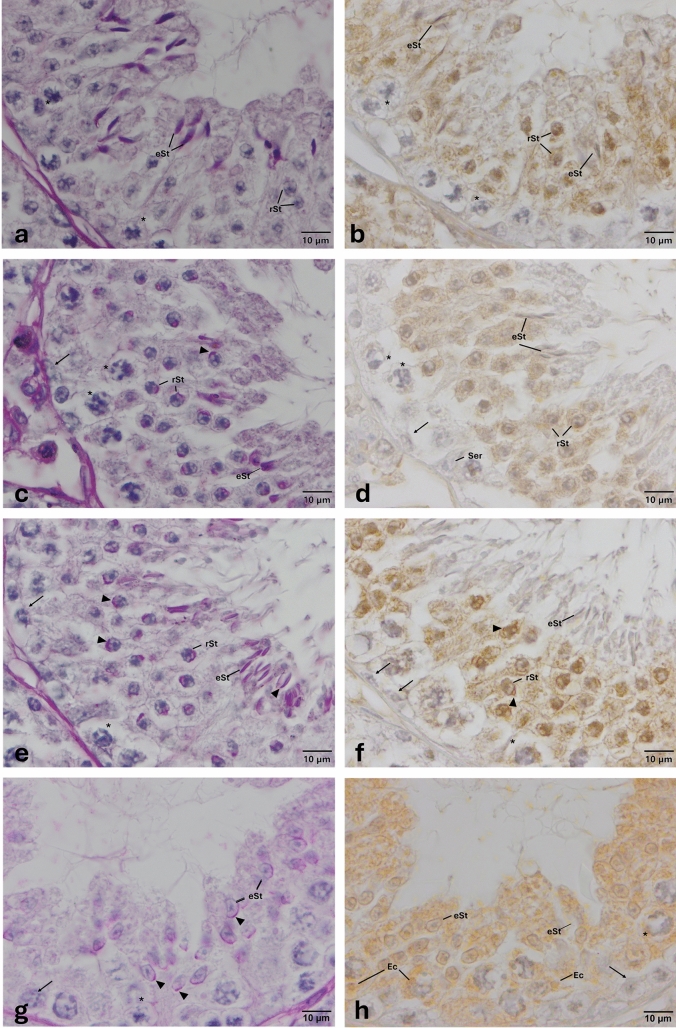
Expression pattern of TLR-1 in the cycle of seminiferous epithelium. Anti- TLR-1 (B-23, Sc-130896, SantaCruz, 1/50) primary antibody was used for detection of cells expressing TLR-1. The presence of round spermatids and/or elongated spermatids with PAS-positive acrosome reveals the cycle of the seminiferous epithelium (arrow heads). **a** Stages I–II; **c** Stages IV–V; **e** Stages VI–VII; and **g** Stages IX–X. Immune-positive cells expressing TLR-1 (**b**, **d** **f**, **h**) appear brown in color. TLR-1 is expressed in spermatocytes, round and elongated spermatids, endosomal compartments, and acrosomes (**b**, **d** **f**, **h**). Please note that TLR-1 is expressed at early, middle, and late stages of spermatogenesis. *eSt* elongated spermatid, *Ec* endosomal compartment, *rSt* round spermatid, *Ser* Sertoli cell, * spermatocytes, and arrows  indicate spermatogonia

**Fig. 2:**
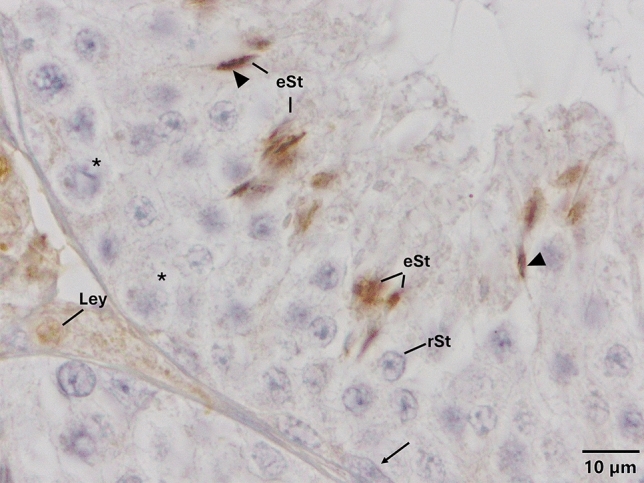
Expression pattern of TLR-2 in the cycle of seminiferous epithelium. Anti-TLR-2 (NB100-56720, Novus, 1/50) primary antibody was used for detection of cells expressing TLR-2. Immune-positive cells expressing TLR-2 appear brown in color. TLR-2 is expressed only in the nuclei and acrosome of elongated spermatids. Please note that PAS staining on the corresponding sequential section (Supplementary Fig. 6) indicates that TLR-2 is expressed at early stage of spermatogenesis. Arrow heads indicate immunopositivity at the acrosome. *eSt* elongated spermatid, *Ley* Leydig cell, *rSt* round spermatid, *spermatocytes, and the arrow indicates spermatogonia

**Fig. 3:**
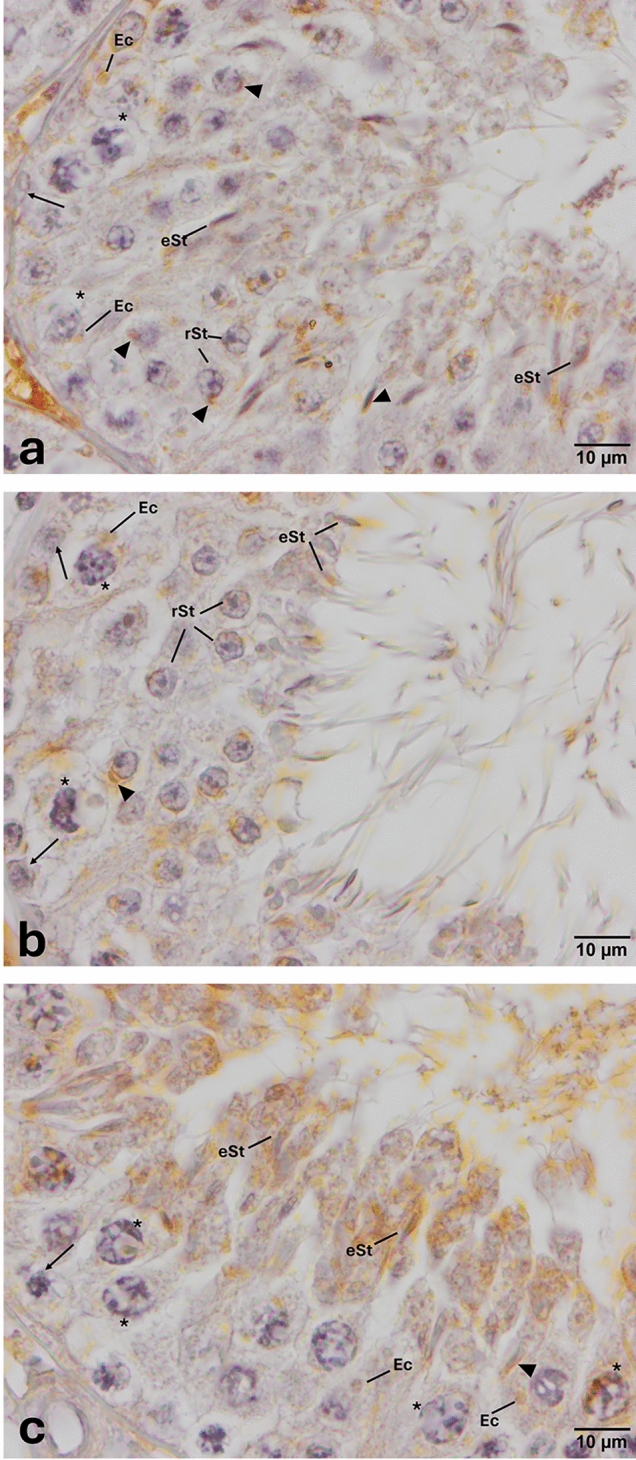
Expression pattern of TLR-3 in the cycle of seminiferous epithelium. Anti-TLR-3 (NB100-56571, Novus, 1/50) primary antibody was used for detection of cells expressing TLR-3. Immune-positive cells expressing TLR-3 (**a**, **b**, **c**) appear brown in color. TLR-3 is expressed in spermatocytes, round and elongated spermatids, endosomal compartments, and acrosomes (**a**, **b**, **c**). Please note that PAS staining on the corresponding sequential section (Supplementary Fig. 7) indicates that TLR-3 is expressed at early, middle, and late stages of spermatogenesis. Arrow heads indicate immunopositivity at the acrosome. *eSt* elongated spermatid, *Ec* endosomal compartment, *rSt* round spermatid, *spermatocytes, and arrows indicate spermatogonia

**Fig. 4:**
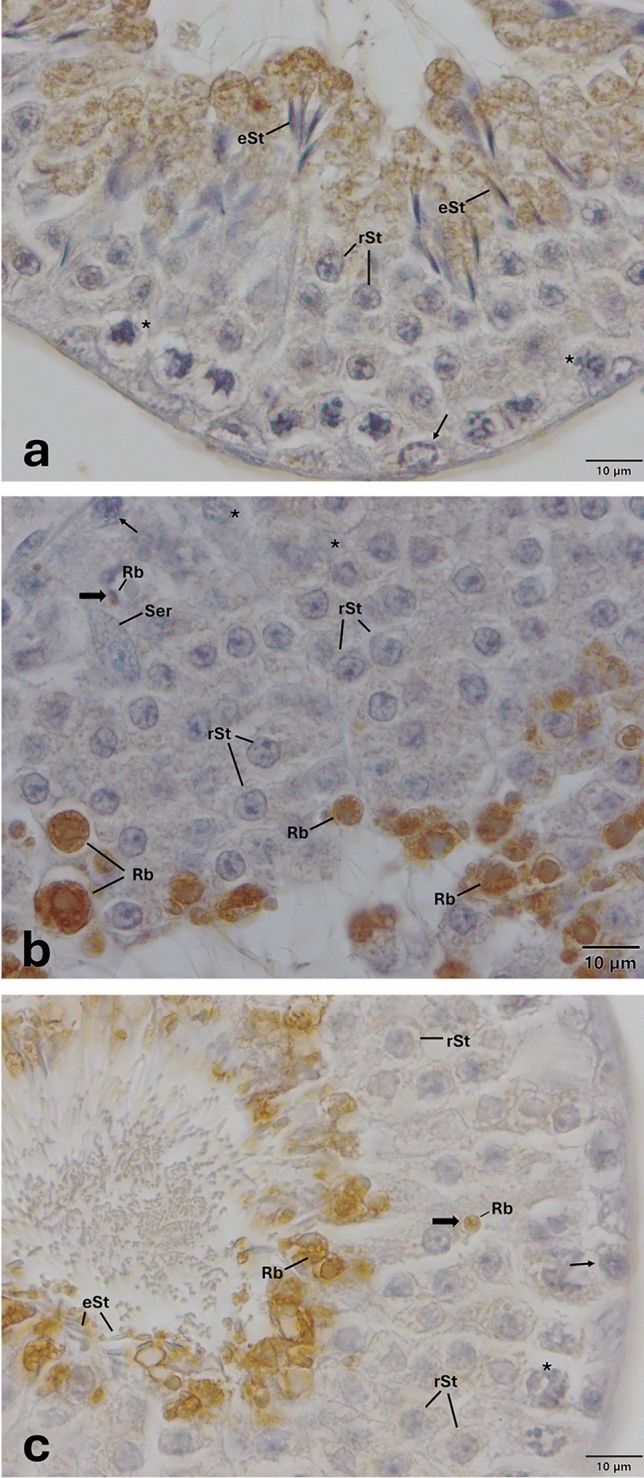
Expression pattern of TLR-4 in the cycle of seminiferous epithelium. Anti-TLR-4 (NB100-56566, Novus, 1/50) primary antibody was used for detection of cells expressing TLR-4. Immune-positive cells expressing TLR-4 (**a**, **b**, **c**) appear brown in color. TLR-4 is expressed only in elongated spermatids and residual bodies (**a**, **b**, **c**). Immune-positive residual bodies are located mainly at the luminal surface of seminiferous of epithelium (**b**, **c**). Please also note the presence of an immune-positive residual body near the nucleus of a Sertoli cell (thick arrow, **b**) and another one close to the basal region of seminiferous of epithelium (thick arrow, **c**). Please note that PAS staining on the corresponding sequential section (Supplementary Fig. 8) indicates that TLR-4 is expressed at early and middle stages of spermatogenesis. *eSt* elongated spermatid, *Rb* residual body, *rSt* round spermatid, *Ser* Sertoli cell, *spermatocytes, and thin arrows indicate spermatogonia

**Fig. 5:**
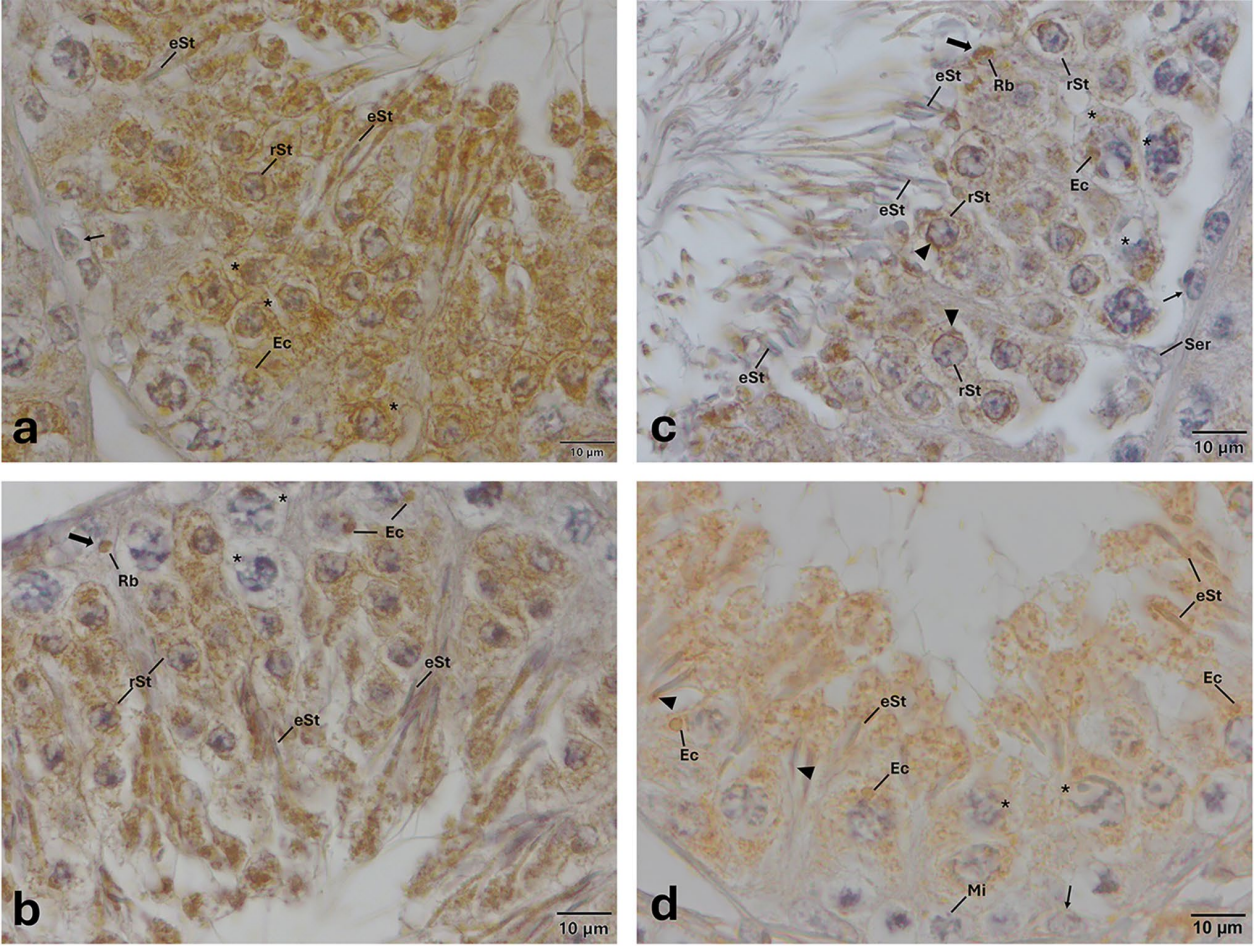
Expression pattern of TLR-5 in the cycle of seminiferous epithelium. Anti-TLR-5 (H-127, Sc-10742, SantaCruz, 1/50) primary antibody was used for detection of cells expressing TLR-5. Immune-positive cells expressing TLR-5 (**a**, **b**, **c**, **d**) appear brown in color. TLR-5 is expressed in spermatocytes, round and elongated spermatids, endosomal compartments, acrosomes, and residual bodies (**a**, **b**, **c**, **d**). Please note the presence of an immune-positive residual body at the basal region of seminiferous of epithelium (thick arrow, **b**), and another one at the luminal surface of seminiferous of epithelium (thick arrow, **c**). Please note that PAS staining on the corresponding sequential section (Supplementary Fig. 9) indicates that TLR-5 is expressed at early, middle, and late stages of spermatogenesis. Arrow heads indicate immunopositivity at the acrosome. *eSt* elongated spermatid, *Ec* endosomal compartment, *Mi* meiosis, *Rb* residual body, *rSt* round spermatid, *Ser* Sertoli cell, *spermatocytes, and thin arrows indicate spermatogonia

**Fig. 6:**
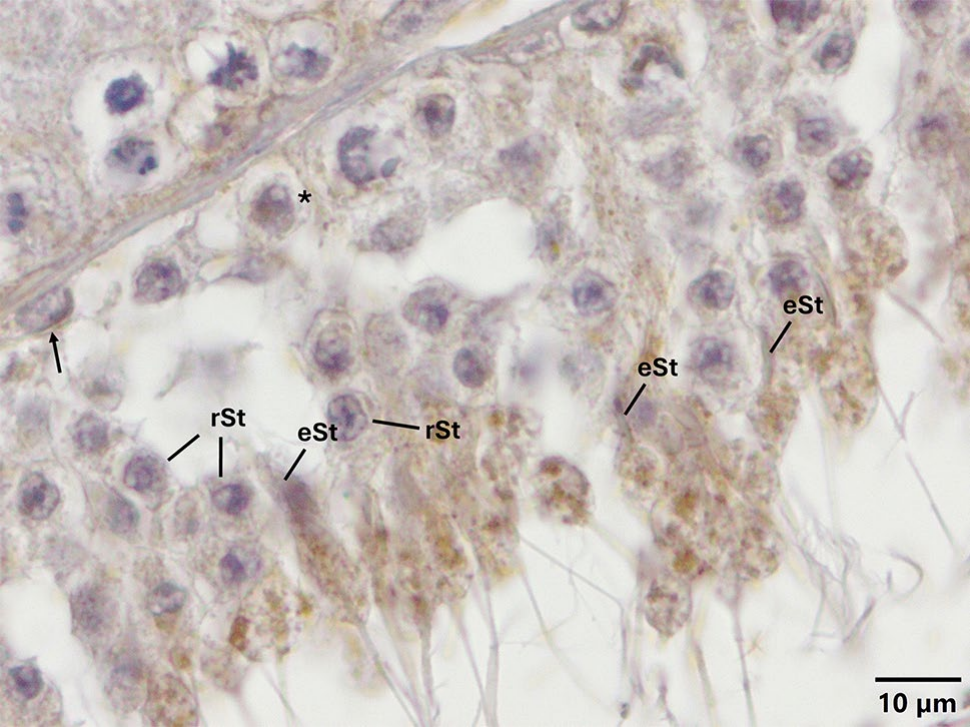
Expression pattern of TLR-7 in the cycle of seminiferous epithelium. Anti-TLR-7 (NB100-56682, Novus, 1/50) primary antibody was used for detection of cells expressing TLR-7. Immune-positive cells expressing TLR-7 appear brown in color. TLR-7 is expressed only in elongated spermatids. Please note that PAS staining on the corresponding sequential section (Supplementary Fig. 10) indicates that TLR-7 is expressed at early stage of spermatogenesis. *eSt* elongated spermatid, *rSt* round spermatid, *spermatocytes, and arrows indicate spermatogonia

**Fig. 7:**
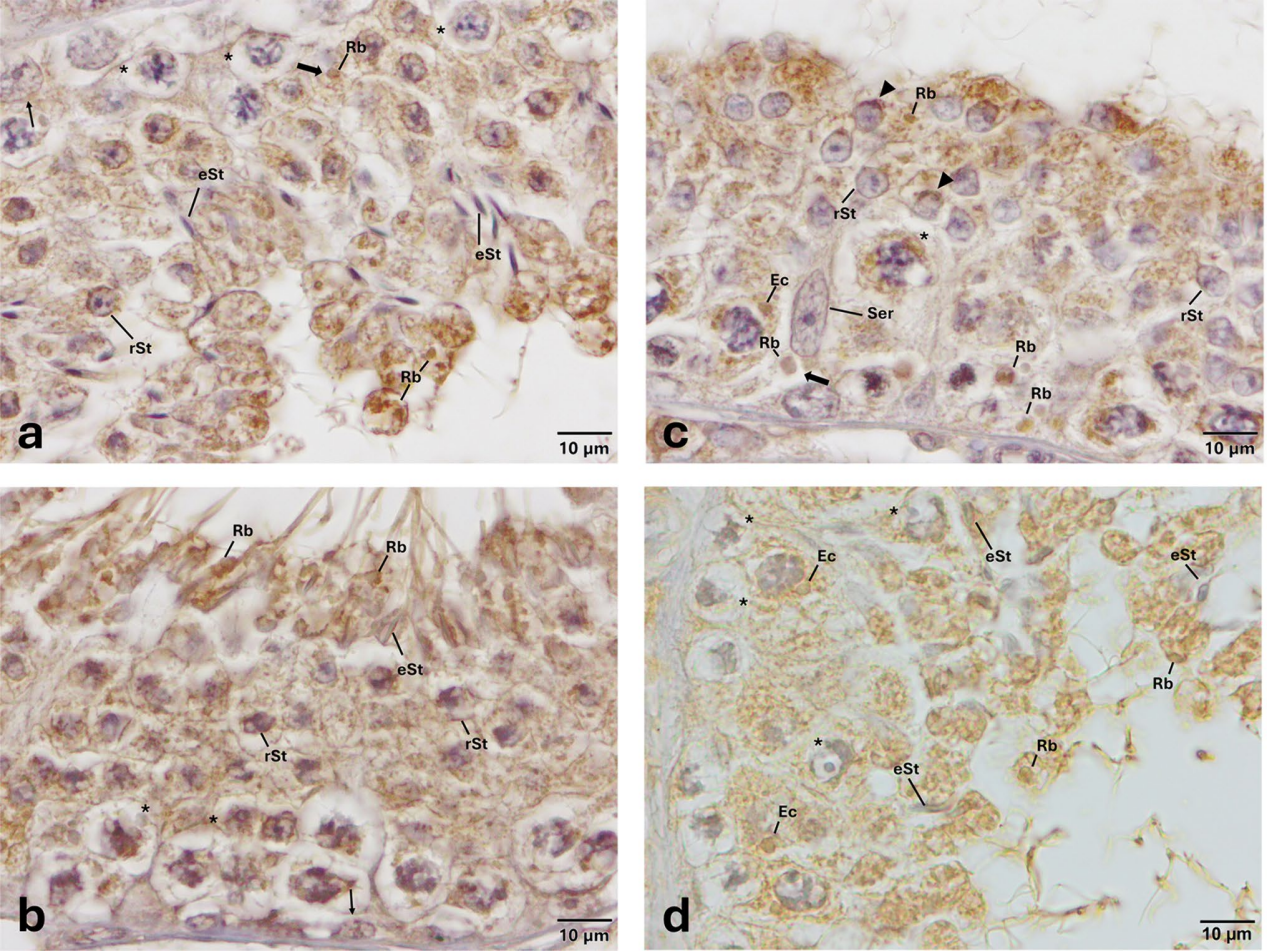
Expression pattern of TLR-11 in the cycle of seminiferous epithelium. Anti-TLR-11 (NBP1-77,204, Novus, 1/50) primary antibody was used for detection of cells expressing TLR-11. Immune-positive cells expressing TLR-11 (**a**, **b**, **c**, **d**) appear brown in color. TLR-11 is expressed in spermatogonia, spermatocytes, round and elongated spermatids, endosomal compartments, acrosomes, and residual bodies (**a**, **b**, **c**, **d**). Immune-positive residual bodies are located at the luminal surface of seminiferous of epithelium (**a**, **b**, **c**, **d**). Please also note the presence of other residual bodies are at the basal region of seminiferous of epithelium (thick arrows, **a**, **c**) and near the nuclei of Sertoli cells (thick arrow, **c**). Please note that PAS staining on the corresponding sequential section (Supplementary Fig. 11) indicates that TLR-11 is expressed at early, middle, and late stages of spermatogenesis. Arrow heads indicate immunopositivity at the acrosome. *eSt* elongated spermatid, *Ec* endosomal compartment, *Rb* residual body, *rSt* round spermatid, *Ser* Sertoli cell, *spermatocytes, and thin arrows indicate spermatogonia

**Fig. 8:**
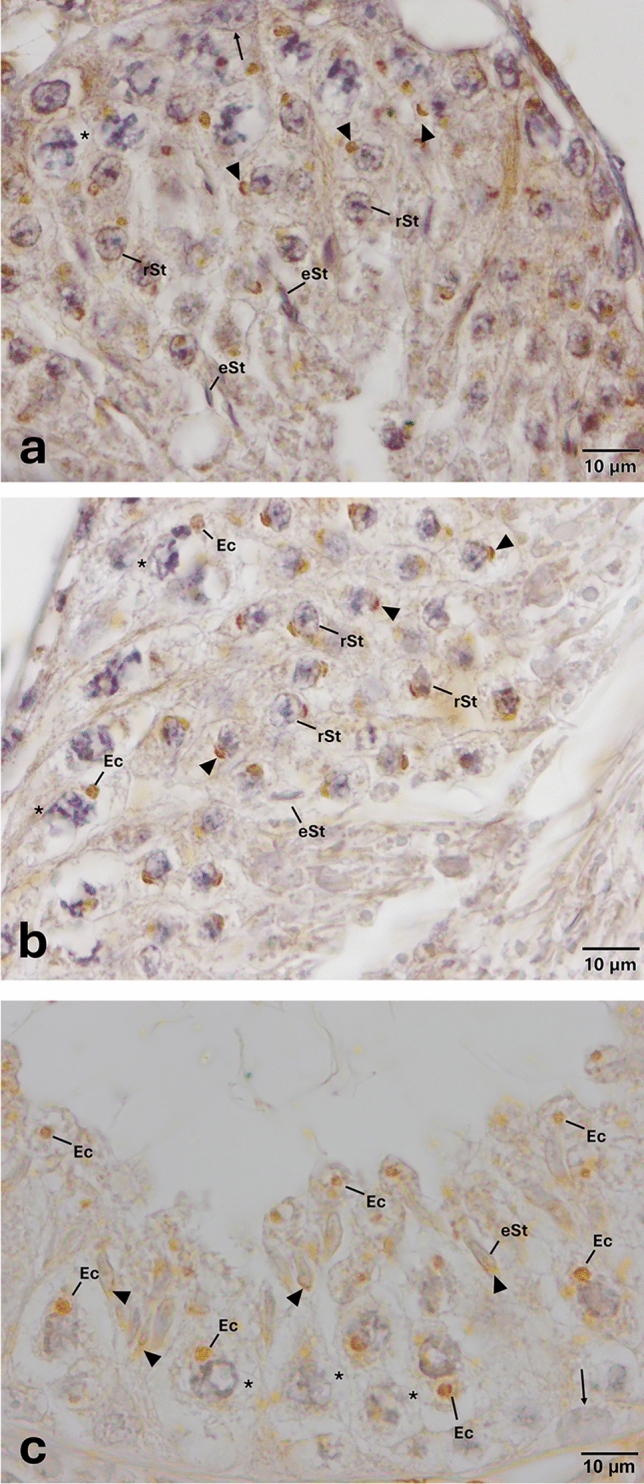
Expression pattern of TLR-12 in the cycle of seminiferous epithelium. Anti-TLR-12 (NBP2-24,833, Novus, 1/50) primary antibody was used for detection of cells expressing TLR-12. Immune-positive cells expressing TLR-12 (**a**, **b**, **c**) appear brown in color. TLR-12 is expressed in spermatocytes, round and elongated spermatids, endosomal compartments and acrosomes (**a**, **b**, **c**). Please note that acrosomes of round spermatids (**a**, **b**), endosomal compartments of both spermatocytes (**b**, **c**) and elongated spermatids (**c**) are immune positive. Please note that PAS staining on the corresponding sequential section (Supplementary Fig. 12) indicates that TLR-12 is expressed at early stage of spermatogenesis. Arrow heads indicate immunopositivity at the acrosome. *eSt* elongated spermatid, *Ec* endosomal compartment, *rSt* round spermatid, *Ser* Sertoli cell, *spermatocytes, and arrows indicate spermatogonia

**Fig. 9:**
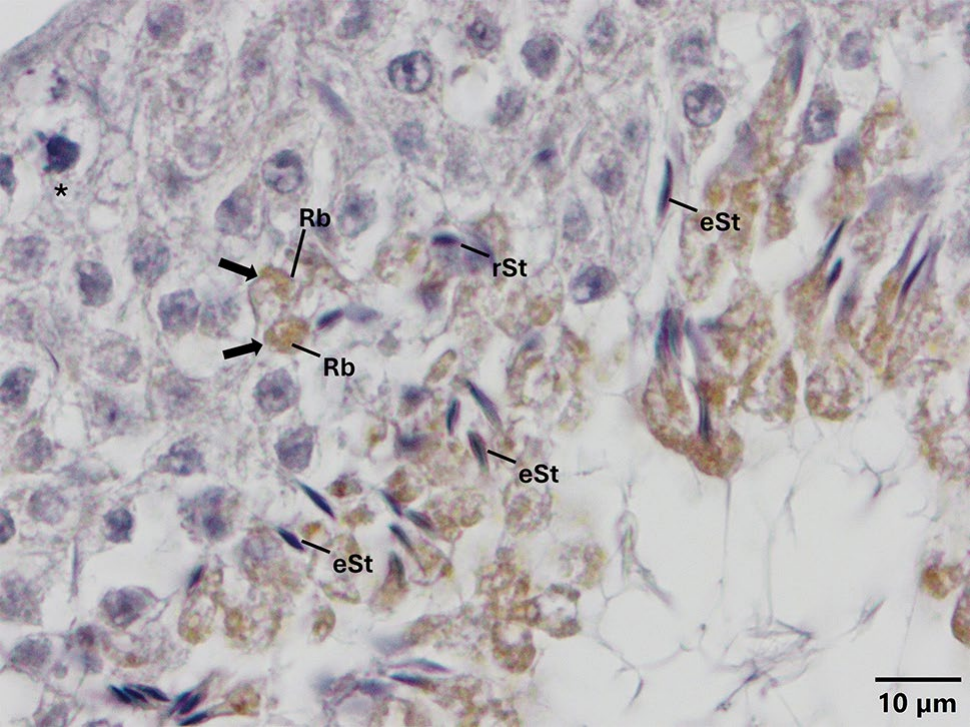
Expression pattern of TLR-13 in the cycle of seminiferous epithelium. Anti-TLR-13 (NBP2-24,539, Novus, 1/50) primary antibody was used for detection of cells expressing TLR-13. Immune-positive cells expressing TLR-13 appear brown in color. TLR-13 is expressed only in elongated spermatids and residual bodies. Please note that the presence of two immune-positive residual bodies located at the luminal surface of the seminiferous of epithelium (arrows). Please note that PAS staining on the corresponding sequential section (Supplementary Fig. 13) indicates that TLR-13 is expressed at early stage of spermatogenesis. *eSt* elongated spermatid, *Rb* residual body, *rSt* round spermatid, *spermatocytes

Microscopic evaluation of the sections at a higher magnification (100×) further revealed that TLR-1, -2, -3, -4, -5, -7, -11, -12, and -13 were expressed by distinct populations of germ cells. Figures 1, 2, 3, 4, 5, 6, 7, 8, and 9 show the expression of TLR-1, -2, -3, -4, -5, -7, -11, -12, and -13, respectively. TLR-1 was expressed by spermatocytes, round, and elongated spermatids. TLR-2, -4, -7, and -13 were only expressed by elongated spermatids. TLR-3, TLR-5, TLR-11, and TLR-12 were expressed by spermatocytes, round, and elongated spermatids, while spermatogonia expressed only TLR-11. Of these, expression of TLR-1, -3, -5, -11, and TLR-12 appeared at endosomal compartments of spermatocytes. TLR-1, -2, -3, -5, -11, and TLR-12 were expressed at the acrosomal complex at round and elongated spermatids. TLR-4, -5, -11, and -13 were specifically expressed in residual bodies either at the luminal surface of the seminiferous epithelium and/or near to the nuclei of Sertoli cells. A summary of the expression of TLRs by the type of germ cells is provided in Table 1.

**Table 1:** Expression of TLRs by the type of germ cells in the adult mouse testis

TLRs	Spermatogonia	Spermatocytes	Round spermatid	Elongated spermatid	Residual body	Endosomal compartment	Acrosome
TLR-1	–	+	+	+	–	+	+
TLR-2	–	–	–	+	–	–	+
TLR-3	–	+	+	+	–	+	+
TLR-4	–	–	–	+	+	–	–
TLR-5	–	+	+	+	+	+	+
TLR-7	–	–	–	+	–	–	–
TLR-11	+	+	+	+	+	+	+
TLR-12	–	+	+	+	–	+	+
TLR-13	–	–	–	+	+	–	–

**Fig. 10:**
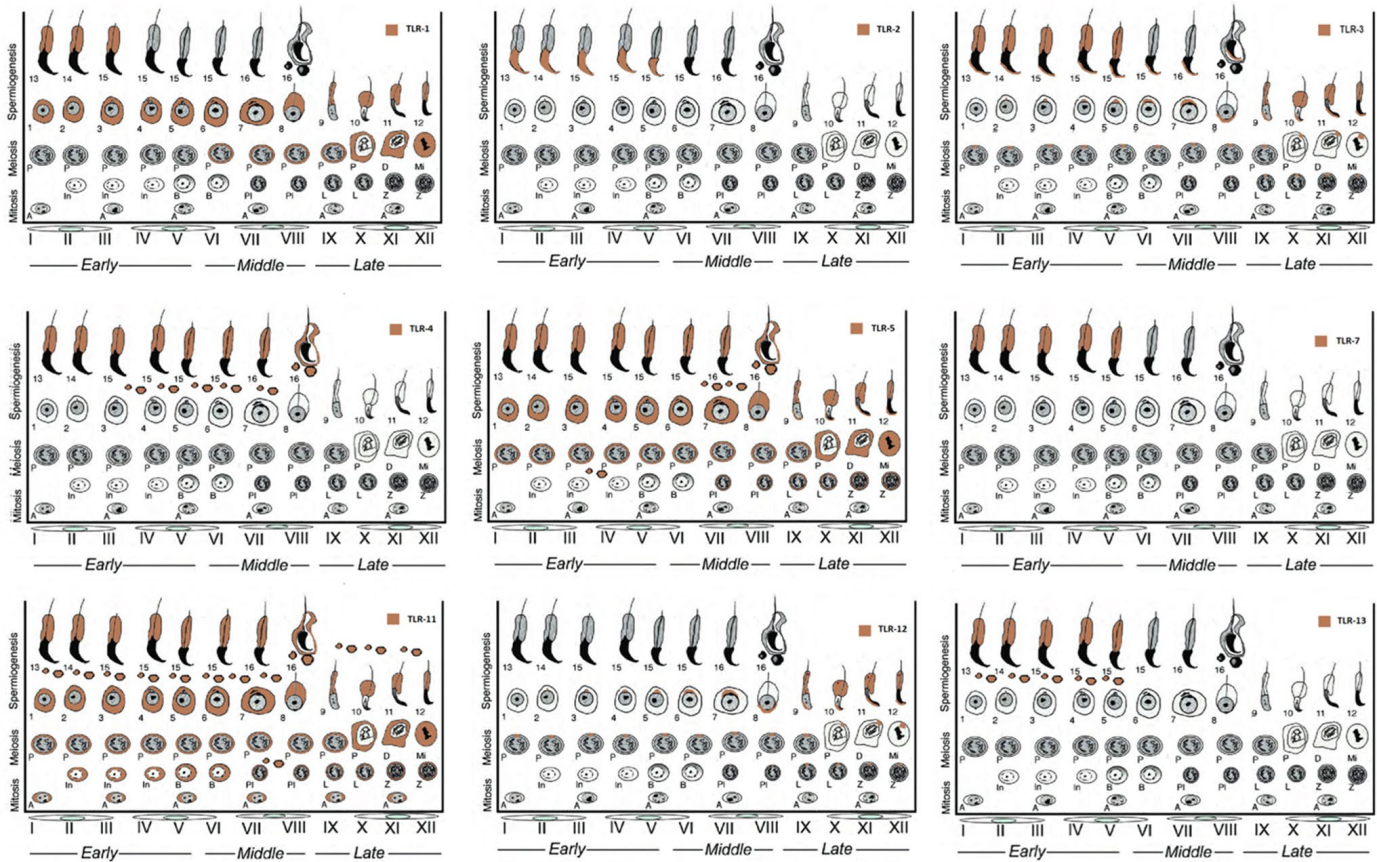
A schematic diagram summarizing the expression of TLR-1, -2, -3, -4, -5, -7, -11, -12 and -13 in the cycle of the seminiferous epithelium. Diagram was modified from Hess and Renato de Franca (2008). Each stage follows one another (stages I–XII). *A*, spermatogonia A; *In*, intermediate spermatogonia; *B*, type B spermatogonia; *Pl* preleptotene, *L* leptotene, *Z* zygotene, *P* pachytene, *D* diakinesis, *Mi* meiotic division; round spermatids (1–8) and elongated spermatids (9–16). Brown color indicates the expression of TLRs by a specific set of germ cells

Results further revealed that the expression of TLR-1, -2, -3, -4, -5, -7, -11, -12, and -13 was not arbitrary and follows a distinct spatiotemporal pattern throughout the cycle of seminiferous epithelium (Figs. 1, 2, 3, 4, 5, 6, 7, 8, 9). PAS staining on sequential section showing the expression of TLR-2, -3, -4, -5, -7, -11, -12, and -13 corresponding to a specific cycle of the seminiferous epithelium is provided in Supplementary Figs. 6–13. TLR-1, -3, -5, -11, and -12 were expressed in all (the early, middle, and late) stages of the spermatogenic cycle. While the expression of TLR-4 was observed at the early and middle stages of spermatogenic cycle, TLR-2, -7, and -13 were expressed only at the early stage in the cycle of the seminiferous epithelium. A summary of the expression of TLRs by germ cells coinciding with a specific cycle of the seminiferous epithelium is provided in Fig. 10.

## Discussion

TLRs are evolutionarily conserved proteins that play an indispensable role in innate immune system by recognizing various PAMPs derived from bacteria, fungi ,and protozoa (Takeda and Akira 2005). In addition to the specific PAMPs, TLRs can also detect endogenous ligands referred to as damage-associated molecular patterns released from damaged or dying cells (DAMPs, Janeway 1989; Yu et al. 2010; Behzadi et al. 2021). Binding of any of these ligands to a specific TLR triggers an innate immune response (Kawai and Akira 2006; Ward 2012), a prerequisite for the killing and clearance of various pathogens (Alexopoulou et al. 2001). In the testis tissue, involvement of TLRs in mediating testicular innate immune response is relatively well established for various somatic cells including Sertoli (Riccioli et al. 2006; Starace et al. 2008; Wu et al. 2008; Sun et al. 2010; Winnall et al. 2011) and Leydig cells (Shang et al. 2011). Nevertheless, to what extent germ cells are involved in this process remains unknown, except for TLR-3 and TLR-11. It was demonstrated that activation of TLR3 through a synthetic double-strained RNA analog leads to increased production of various proinflammatory cytokines and antiviral proteins in germ cells (Wang et al. 2012). Similarly, Chen et al. (2014) demonstrated in the mouse that activation of TLR-11 by *Toxoplasma gondii*-derived profilin and uropathogenic *Escherichia coli* (UPEC) can induce an innate immune response in germ cells through the production of inflammatory cytokines. In light of evidence provided in the present study demonstrating the expression of all functional TLRs in various populations of germ cells, further investigations are warranted to obtain a better and more comprehensive understanding of the role of each TLRs in modulating testicular innate immune response.

There is ample evidence demonstrating that all TLRs, except for TLR-13, are expressed by male germ cells (Bhushan et al. 2008; Wang et al. 2012; Chen et al. 2014; Saeidi et al. 2014; Nejsum et al. 2019; Sun et al. 2019; Özbek et al. 2020; Öztop et al. 2020). However, in the mouse, with the model used in the present study, the expression pattern of TLRs by specific populations of germ cells has only been demonstrated for TLR-3 (Wang et al. 2012; Nejsum et al. 2019), TLR-9 (Mihara et al. 2010) and TLR-11 (Chen et al. 2014). Accordingly, spermatogonia and spermatocytes express both TLR-3 and TLR-11 (Wang et al. 2012; Chen et al. 2014; Nejsum et al. 2019). While TLR-11 is also expressed by spermatids (Chen et al. 2014), TLR-9 is expressed only by spermatozoa (Mihara et al. 2010). While confirming the expression of TLR-11 by spermatogonia, spermatocytes, and spermatids as well as the expression of TLR-3 by spermatocytes, results of the present study further reveal the expression pattern of the remaining functional TLRs by specific populations of germ cells. It appears that, on top of TLR-3 and -11, spermatocytes also express TLR-1, -5, and -12. It is also evident that the expression of TLRs by spermatids is not limited to TLR-11. While TLR-1, -3, -5, and -12 are expressed by round, and elongated spermatids, elongated spermatids express only TLR-2, -4, -7, and -13. To the best of our knowledge, the present study is the first revealing the expression pattern of all functional TLRs simultaneously by germ cells in the mouse testis throughout the cycle of the seminiferous epithelium from stage I to XII. It is evident from these observations that throughout spermatogenesis TLRs are differentially expressed by various populations of germ cells.

Expression of TLR-1, -3, -5, -11, and -12 at endosomal compartments of elongated spermatids (Fig. 8) and spermatocytes (Figs. 1, 3, 5, 7, 8) and confinement of the expression of TLR-1, -2, -3, -5, -11 and -12 (Figs. 1, 2, 3, 5, 7, 8) to the acrosomes of round and/or elongated spermatids are the two most interesting and novel findings of the present study. TLRs are synthesized in the endoplasmic reticulum (ER), transported to the Golgi bodies, and finally travel either to the cell surface (TLR-1–6 and -10) or stay in endosomes and/or lysosomes (TLR-3, -7, -8, -9, -11, -12, and -13) (Kawai and Akira 2010; Celhar et al. 2012; Kawasaki and Kawai 2014; Lee and Barton 2014). In light of evidence suggesting that endosomes might also give rise to the acrosome (Martínez-Menárguez et al. 1996; Sun-Wada et al. 2002; Moreno and Alvarado, 2006), results of the present study revealing to the best of our knowledge for the first time, the expression of specific TLRs at endosomal compartments and/or acrosome appear to be coherent with the synthesis and trafficking of TLRs. However, whether or not TLRs are differentially expressed by specific subsets of endosomal compartments such as early endosomes (Lakadamyali et al. 2006), recycling endosomes (Rink et al. 2005), late endosomes/multivesicular bodies (Russell et al. 2006), and lysosomes (Stein et al. 2003) warrant further investigations. Another interesting finding of the present study was the expression of TLR -4, -5, -11, and -13 at residual bodies, composed of various organelles, such as Golgi complex and ER, that the sperm cell no longer needs (de Kretser and Kerr 1988). Residual bodies are the cytoplasmic fragments of late spermatids which are removed at the time of sperm release (Syed et al. 1995). When spermatids are released into the lumen of the seminiferous epithelium, residual bodies are phagocytosed by Sertoli cells (O’Donnell et al. 2011), transported to the basal compartment, and catabolized (Johnson 2015). Lysosomes of Sertoli cells then fuse with the residual bodies to form phagolysosomes (de Kretser and Kerr 1988) resulting in phagocytosis of the residual bodies of germ cells (Wu et al. 2008; Chojnacka et al. 2016; Chen et al. 2020). In light of the fact that phagocytosis of residual bodies by Sertoli cells is an essential process for spermatogenesis (Wu et al. 2008; Li et al. 2012; Chojnacka et al. 2016; Chen et al. 2020), details surrounding the role of TLR -4, -5, -11, and -13 in this process warrant more in-depth studies. It is well established that activation of TLRs facilitates phagosome maturation (Blander and Medzhitov 2004) and is involved in the activation of autophagy (Xu et al. 2007). It is also interesting to note in this context that the removal of damaged and/or dysfunctional mitochondria in the residual bodies by mitophagy, viz. selective degradation of mitochondria by autophagy, is a critical process for the generation of individual spermatozoa and proper rearrangement of mitochondria (Sakai and Yamashina 1989; Ho and Wey 2007; Huang et al. 2021). Whether or not TLRs expressed at residual bodies of spermatids are involved in any of these processes remains to be determined. In any case, if and to what extent expression of specific TLRs at endosomal compartments, acrosomes, and/or residual bodies play in the regulation of the cycle of spermatogenesis remains an open question.

Evidence gathered in the present study demonstrating differential expression of TLR-1, -2, -3, -4, -5, -7, -11, -12, and -13 by germ cells in accordance with the cycle of the seminiferous epithelium is arguably the most important finding of the present study. While TLR-1, -2, -3, -4, -5, -7, -11, -12, and -13 were expressed at the early (I–V) stages, TLR-1, -3, -4, -5, -11, and -12 were expressed at the middle (VI–VIII) stages in the spermatogenic cycle of the seminiferous epithelium. On the other hand, TLR-1, -3, -5, -11, and -12 were expressed at the late (IX–XII) stages. To the best of our knowledge, this is the first study demonstrating the spatiotemporal expression of all functional TLRs throughout the cycle of the seminiferous epithelium in a stage-specific manner. How stage-specific expression of TLRs is regulated remains elusive. In light of the fact that developing spermatogenic cells produce various autoantigens after gaining the ability to generate an immune response (Yule et al. 1988; Zhao et al. 2014), it is tempting to speculate that stage-specific expression of TLRs might be involved in preventing immune response to germ cell-specific as well as paternal major histocompatibility complex (MHC) antigens (Zhao et al. 2014). To what extent this process is associated with the immune-privileged status of the testis tissue also warrants further investigations (Head and Billingham 1985; Fijak et al. 2011). Considering that apoptosis is a physiological process of spermatogenesis (Nakanishi and Shiratsuchi 2004; Zhao et al. 2014; Zakariah et al. 2022) and that some members of the TLR family are capable of inducing apoptosis (Aliprantis et al. 2000; Salaun et al. 2006), TLRs might also be involved in the regulation of germ cell apoptosis. Furthermore, there is a substantial body of evidence indicating that, apart from their immune functions, TLRs play a role in various developmental processes including the regulation of neurogenesis (Rolls et al. 2007) and aging (Okun et al. 2009). In any case, whether or not the expression of TLRs by specific populations of germ cells in a cycle-dependent manner has any non-immune and/or developmental function(s) in the regulation of spermatogenesis remains to be determined.

Taken together, results of the present study strengthen the hypothesis that the expression of TLRs by male germ cells is a developmentally regulated process and point to their possible involvement in the regulation of testicular functions (Hedger 2011) and spermatogenesis (Girling and Hedger 2007). Nevertheless, specific function of each TLR in sequential stages of proliferation, growth, maturation, and differentiation of germ cells throughout the cycle of the seminiferous epithelium remains elusive and warrants further investigations.

### Supplementary Information

Below is the link to the electronic supplementary material.

Supplementary file1 (PDF 2628 KB)

## Data Availability

A more detailed description of immunohistochemistry protocols used in the present study is openly available at https://karger.com/cto/article-abstract/doi/10.1159/000529974/842161/Expression-of-Toll-Like-Receptors-in-the-Lung?redirectedFrom=fulltext. Further inquiries can be directed to the corresponding author.
