# Supplementary material for: Stage-specific expression of Toll-like receptors in the seminiferous epithelium of mouse testis
Source: Histochem Cell Biol. 2024 Jul 31;162(4):323–35. doi: 10.1007/s00418-024-02310-z (PMC11364606; doi:10.1007/s00418-024-02310-z)
Supplement: Supplementary file 1 — Supplementary file1 (PDF 2628 KB) [file 418_2024_2310_MOESM1_ESM.pdf]

Stage-specific expression of toll-like receptors in the seminiferous epithelium of mouse testis

Histochemistry and Cell Biology

Göksel Doğan[1], Mustafa Sandıkçı[1], Levent Karagenç[1]*

[1]Adnan Menderes University, Faculty of Veterinary Medicine, Department of Histology-Embryology, Aydın, 09000, Turkey.

* Corresponding Author: Levent Karagenç

* Adnan Menderes University, Faculty of Veterinary Medicine, Department of Histology-Embryology, 09000, Aydın, TURKEY.

E-mail: lkaragenc@adu.edu.tr

Göksel Doğan https://orcid.org/0000-0002-4583-3140

Mustafa Sandıkçı https://orcid.org/0000-0002-9126-1016

Levent Karageç https://orcid.org/0000-0003-2074-2450

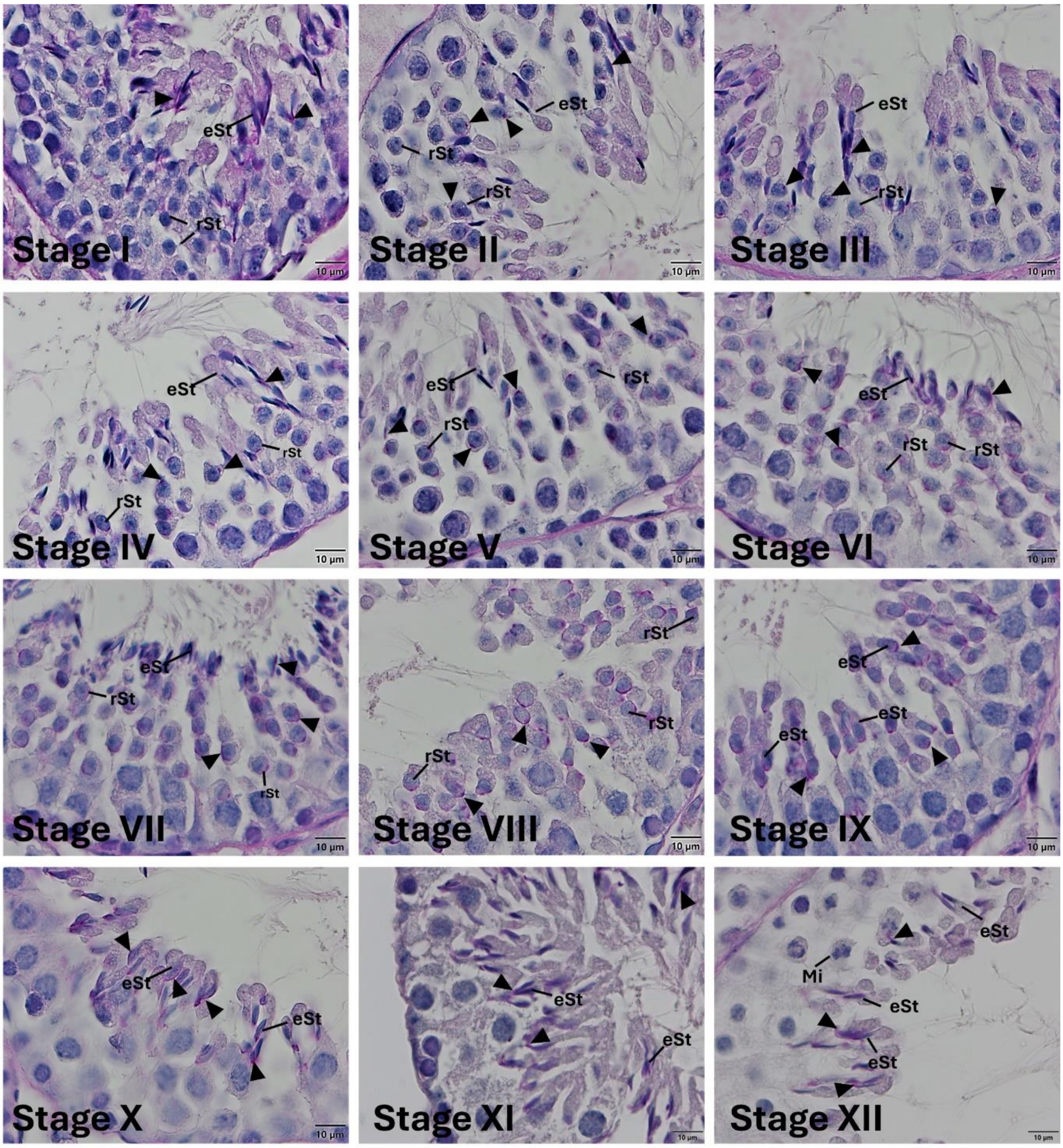

Supplementary Fig. 1 Cycle of the seminiferous epithelium in the mouse. PAS staining was used to define each stage, from Stage I to XII. Sections were counterstained with hematoxylin. Please note that the presence of round spermatids and/or elongated spermatids with PAS-positive acrosomal caps reveals the cycle of the seminiferous epithelium (arrow heads). Please also note that round spermatids lack PAS positive granular material at Stage I. eSt: elongated spermatid; rSt: round spermatid.

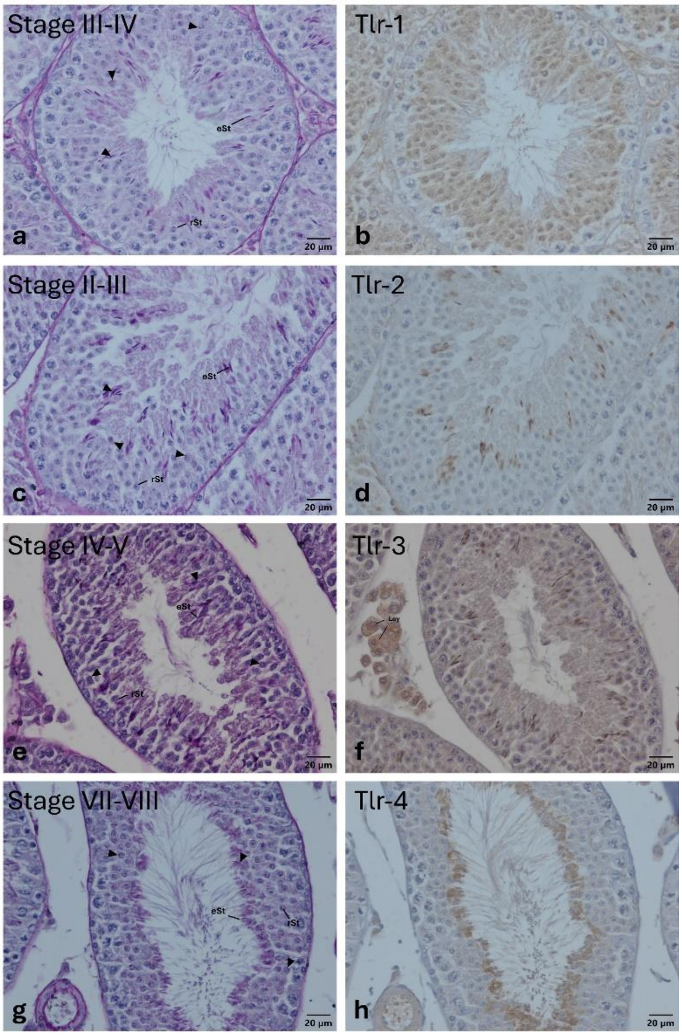

Supplementary Fig. 2 Expression pattern of TLR-1, - 2, -3, and -4 in the cycle of seminiferous epithelium. In order to demonstrate the expression pattern of TLR-1, -2, -3, -4 throughout the cycle of seminiferous epithelium Periodic acid Schiff (PAS) staining and immunohistochemistry were used in 5µm sequential sections. PAS staining was used to define the stages of the seminiferous epithelium. Immunohistochemistry was used to detect cells expressing TLR-1, - 2, -3, and -4 using anti- TLR-1 (B-23, Sc-130896, SantaCruz, 1/50), anti-TLR-2 (NB100-56720, Novus, 1/50), anti-TLR-3 (NB100-56571, Novus, 1/50), anti-TLR-4 (NB100-56566, Novus, 1/50) primary antibodies. Sections were counterstained with hematoxylin. The presence of round spermatids and/or elongated spermatids with PAS-positive acrosomal caps reveals the cycle of the seminiferous epithelium (arrow heads). **a** Stage III-IV; **c** Stage II-III; **e** Stage IV-V; **g**; Stage VII-VIII. Immune-positive cells expressing TLR-1 (**b**), TLR-2 (**d**), TLR-3 (**f**) and TLR-4 (**h**) appear brown in color. TLR-1, TLR-2, TLR-3 and TLR-4 are expressed in germ cells (**b, d, f, h**). TLR-3 is also expressed by Leydig cells. eSt: elongated spermatid; Ley: Leydig cell; rSt: round spermatid.

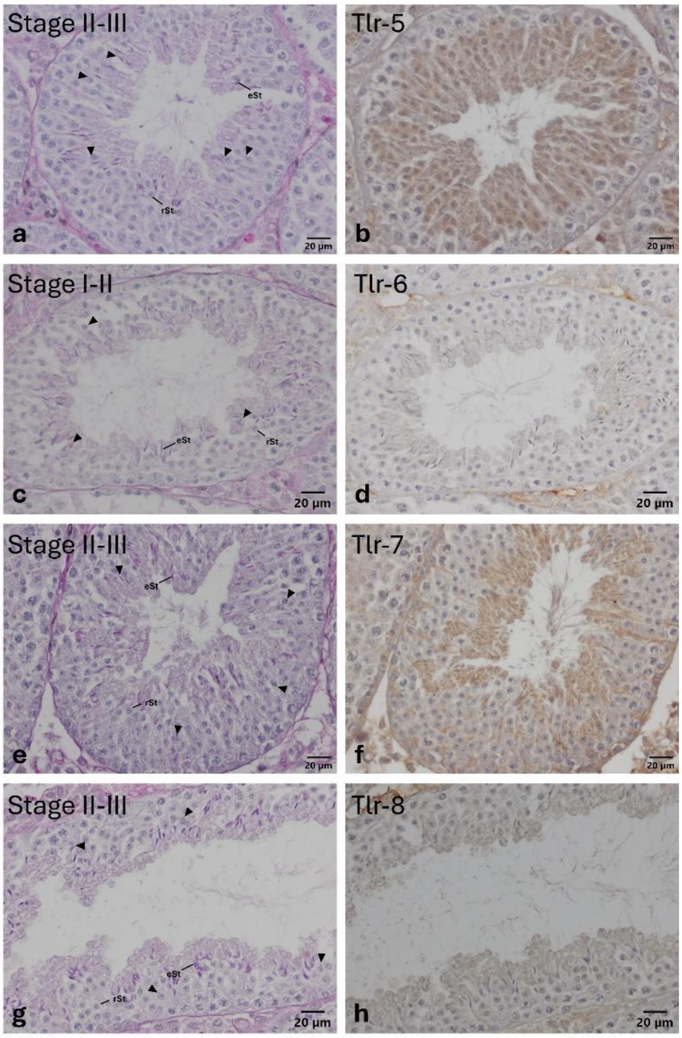

Supplementary Fig. 3 Expression pattern of TLR-5 and -7 in the cycle of seminiferous epithelium. Anti-TLR-5 (H-127, Sc-10742, SantaCruz, 1/50), anti-TLR-6 (NBP1-54336, Novus, 1/50), anti-TLR-7 (NB100-56682, Novus, 1/50), anti-TLR-8 (NBP2-24917, Novus, 1/50) primary antibodies were used for detection of cells expressing TLR-5, -6, -7 and -8. The presence of round spermatids and/or elongated spermatids with PAS-positive acrosomal caps reveals the cycle of the seminiferous epithelium (arrow heads). **a** Stage II-III; **c** Stage I-II; **e** Stage II-III; **g** Stage II-III. Immune-positive cells expressing TLR-5 (**b**) and TLR-7 (**f**) appear brown in color. While TLR-5 and -7 are expressed by germ cells (**b , f**), no expression of TLR-6 and -8 was observed (**d, h**). eSt: elongated spermatid; rSt: round spermatid.

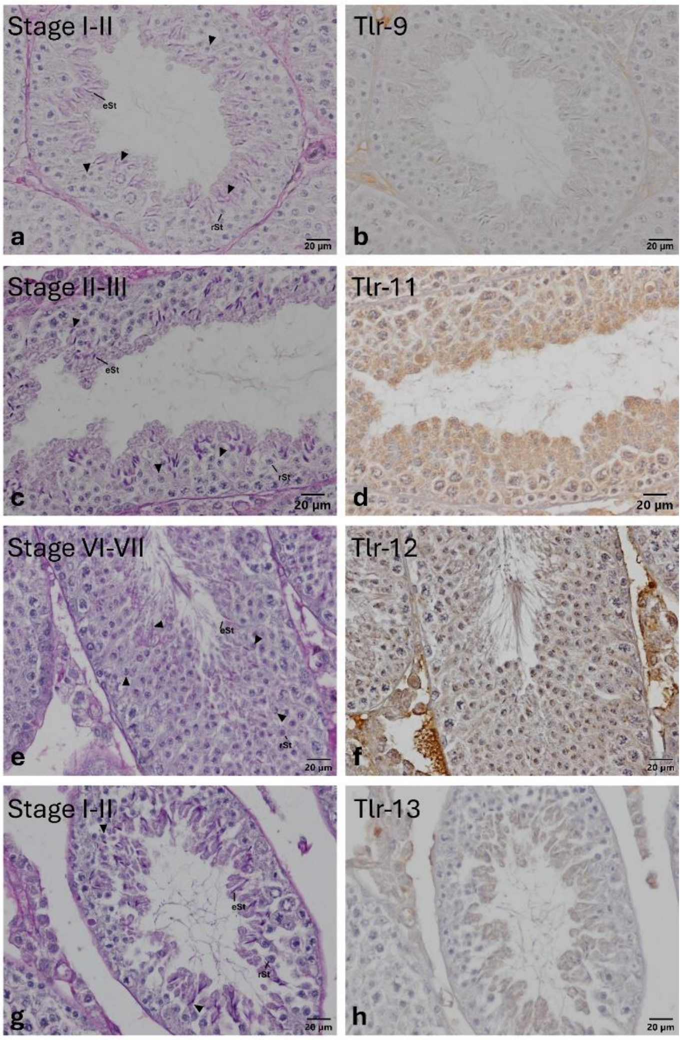

Supplementary Fig. 4 Expression pattern of TLR-11, -12 and -13 in the cycle of seminiferous epithelium. Anti-TLR-9 (NBP2-24729, Novus, 1/50), anti- TLR-11 (NBP1-77204, Novus, 1/50), anti-TLR-12 (NBP2-24833, Novus, 1/50), anti-TLR-13 (NBP2-24539, Novus, 1/50) primary antibodies were used for detection of cells expressing TLR-9, -11, -12, and -13. The presence of round spermatids and/or elongated spermatids with PAS-positive acrosomal caps reveals the cycle of the seminiferous epithelium (arrow heads). **a** Stage I-II; **c** Stage II-III; **e** Stage VI-VII; **g** Stage I-II. Immune-positive cells expressing TLR-11 (**d**), -12 (**f**) and -13 (**h**) appear brown in color. While TLR-11, -12, and -13 are expressed by germ cells (**d, f, h**) no expression of TLR-9 was observed (**b**). eSt: elongated spermatid; rSt: round spermatid.

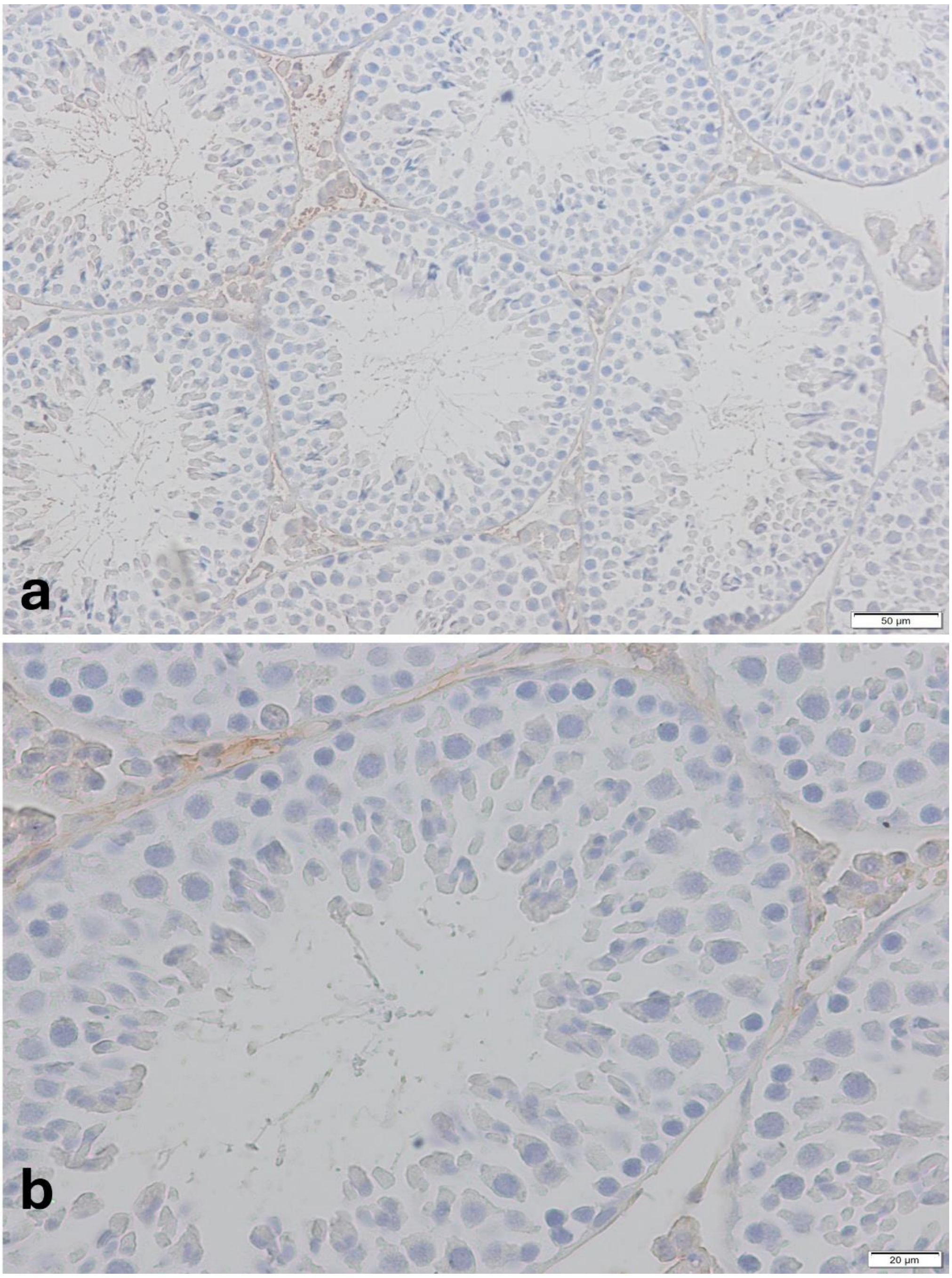

Supplementary Fig. 5 The negative control sections were treated in an identical manner except for the use of TBS (pH 7.6) instead of the primary antibodies. After a final rinse and wash in TBS, immune positive cells were detected using 3, 3’ diaminobenzidine tetrahydrochloride (DAB) solution (3 mg/ml in Tris-HCl, pH 7,6 with 3 % H2O2). Sections were counterstained with Mayer’s hematoxylin. The sections were observed on an Olympus BX51 microscope and images were captured using Olympus DP70 camera with DP controller software (Ver. 3.1.1.267). No immune-positivity were detected in any of the negative control sections used for each antibody in testis tissues. **a** 20X magnification; **b** 40X magnification.

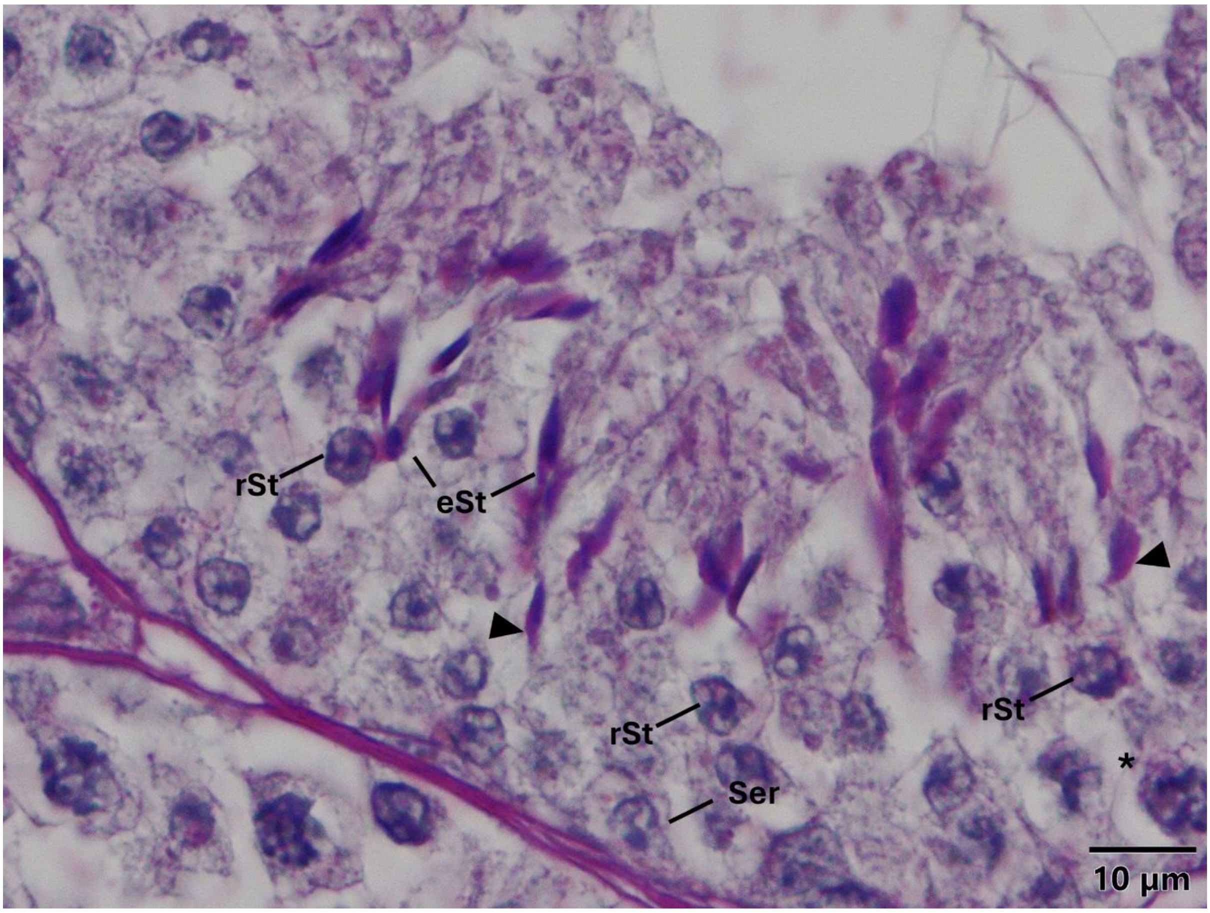

Supplementary Fig. 6 PAS staining on a sequential section for TLR-2 demonstrating the cycle of the seminiferous epithelium. The presence of round spermatids and/or elongated spermatids with PAS-positive acrosome reveals the cycle of the seminiferous epithelium (arrow heads). Stage I-II. eSt: elongated spermatid; rSt: round spermatid; Ser: Sertoli cell; *: spermatocytes.

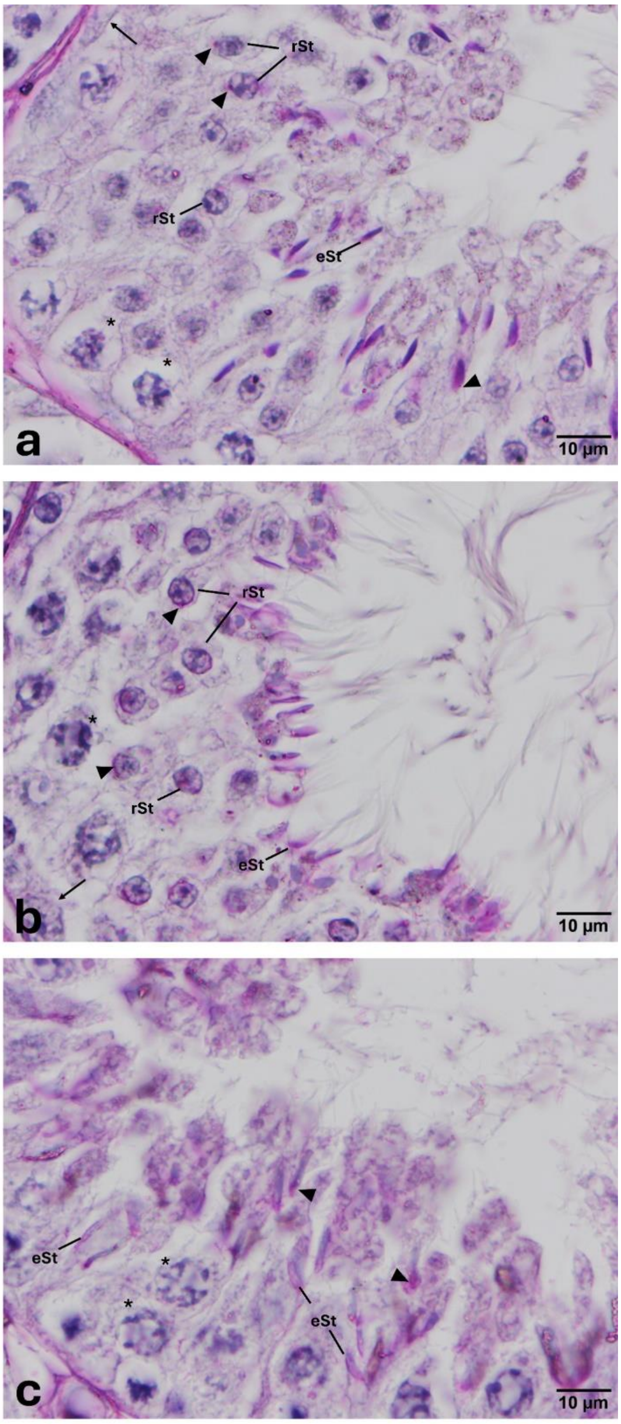

Supplementary Fig. 7 PAS staining on a sequential section for TLR-3 demonstrating the cycle of the seminiferous epithelium. The presence of round spermatids and/or elongated spermatids with PAS-positive acrosome reveals the cycle of the seminiferous epithelium (arrow heads). **a** Stage III-IV; **b** Stage VII-VIII; **c** Stage XI-XII. eSt: elongated spermatid; rSt: round spermatid; *: spermatocytes; arrows; SG: spermatogonia.

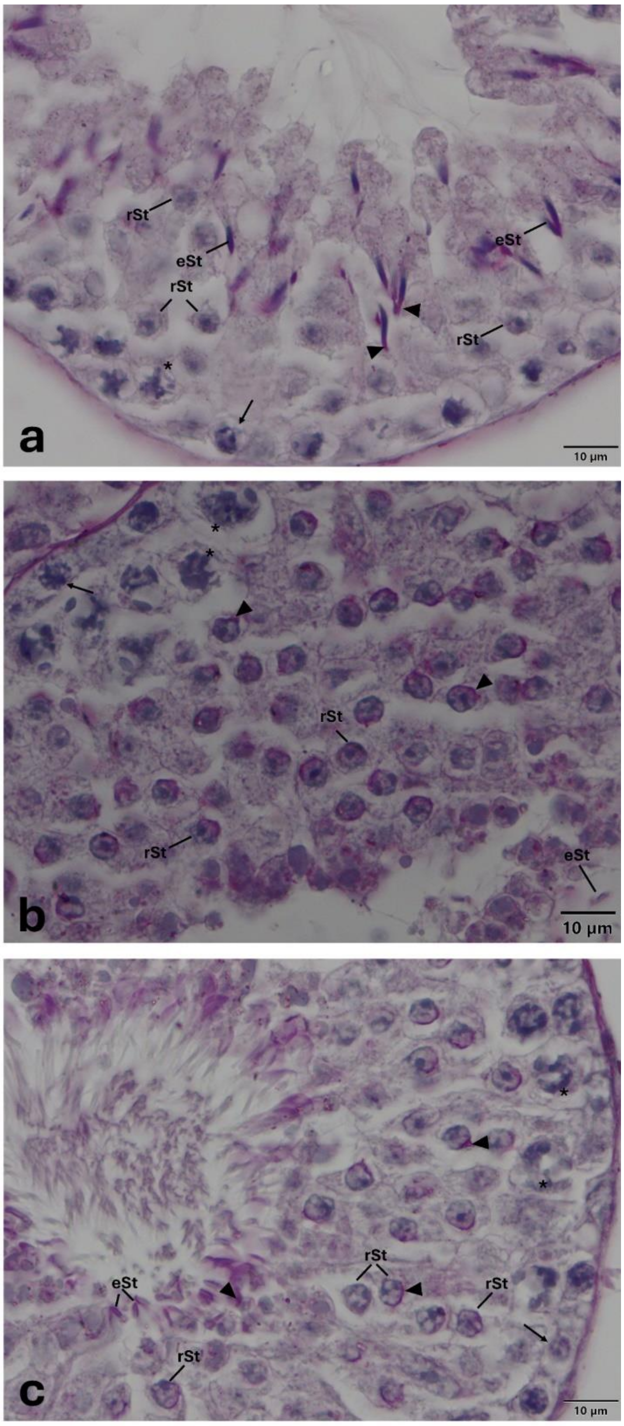

Supplementary Fig. 8 PAS staining on a sequential section for TLR-4 demonstrating the cycle of the seminiferous epithelium. The presence of round spermatids and/or elongated spermatids with PAS-positive acrosome reveals the cycle of the seminiferous epithelium (arrow heads). **a** Stage I-II; **b** Stage VI-VII; **c** Stage VII-VIII. eSt: elongated spermatid; rSt: round spermatid; *: spermatocytes; arrows: spermatogonia.

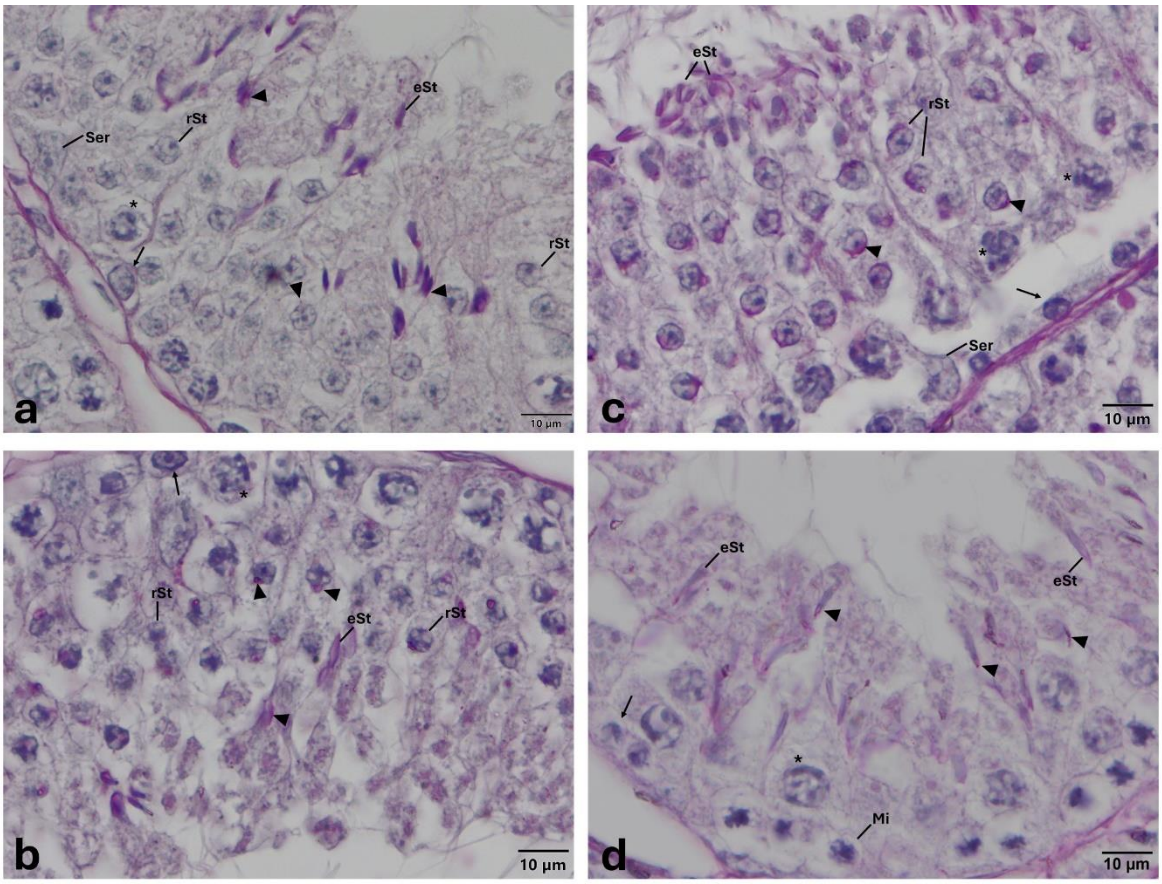

Supplementary Fig. 9 PAS staining on a sequential section for TLR-5 demonstrating the cycle of the seminiferous epithelium. The presence of round spermatids and/or elongated spermatids with PAS-positive acrosome reveals the cycle of the seminiferous epithelium (arrow heads). **a** Stage I-II; **b** Stage III-IV; **c** Stage VI-VII; **d** Stage XI-XII. eSt: elongated spermatid; Mi: meiosis; rSt: round spermatid; Ser: Sertoli cell; *: spermatocytes; arrows: spermatogonia.

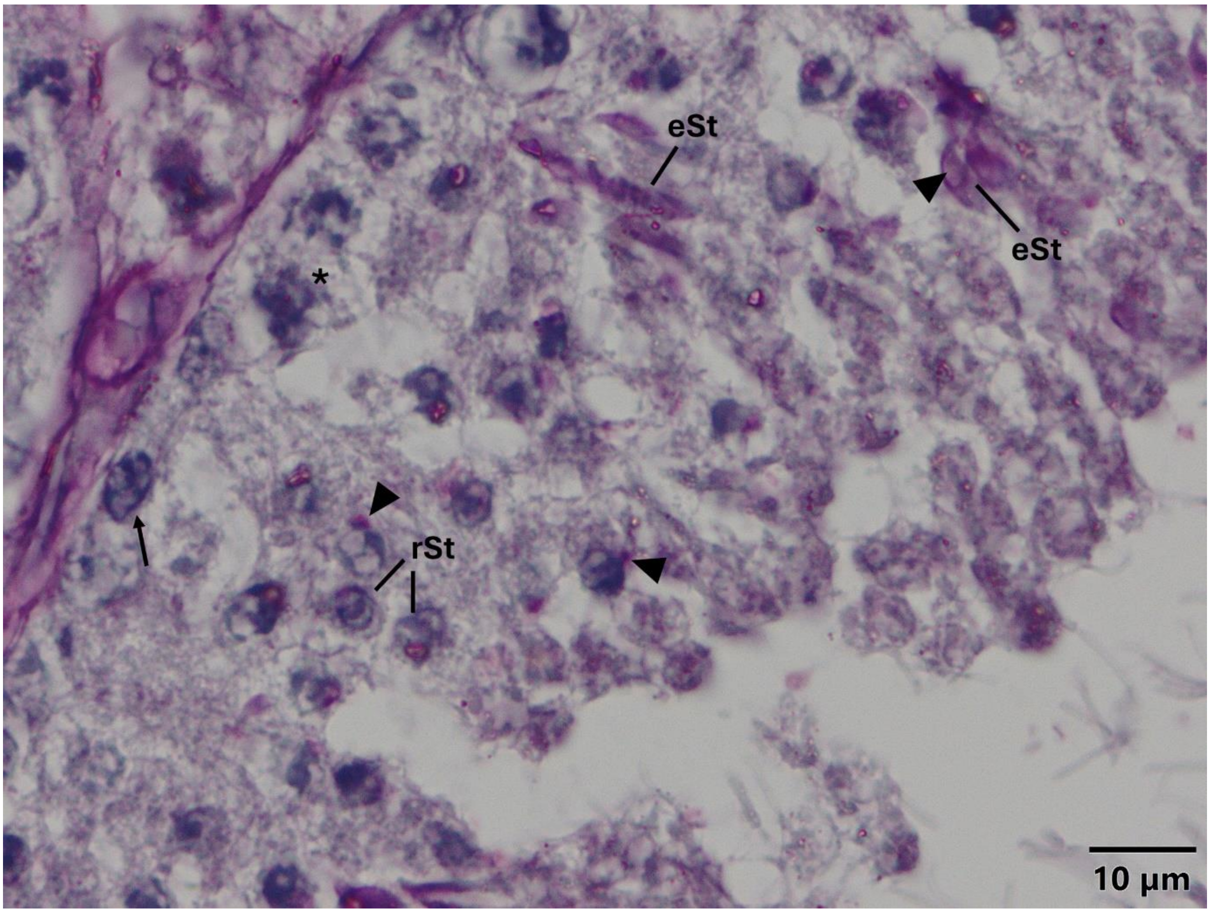

Supplementary Fig. 10 PAS staining on a sequential section for TLR-7 demonstrating the cycle of the seminiferous epithelium. The presence of round spermatids and/or elongated spermatids with PAS-positive acrosome reveals the cycle of the seminiferous epithelium (arrow heads) at stage III-IV. eSt: elongated spermatid; rSt: round spermatid; *: spermatocytes; arrow: spermatogonia.

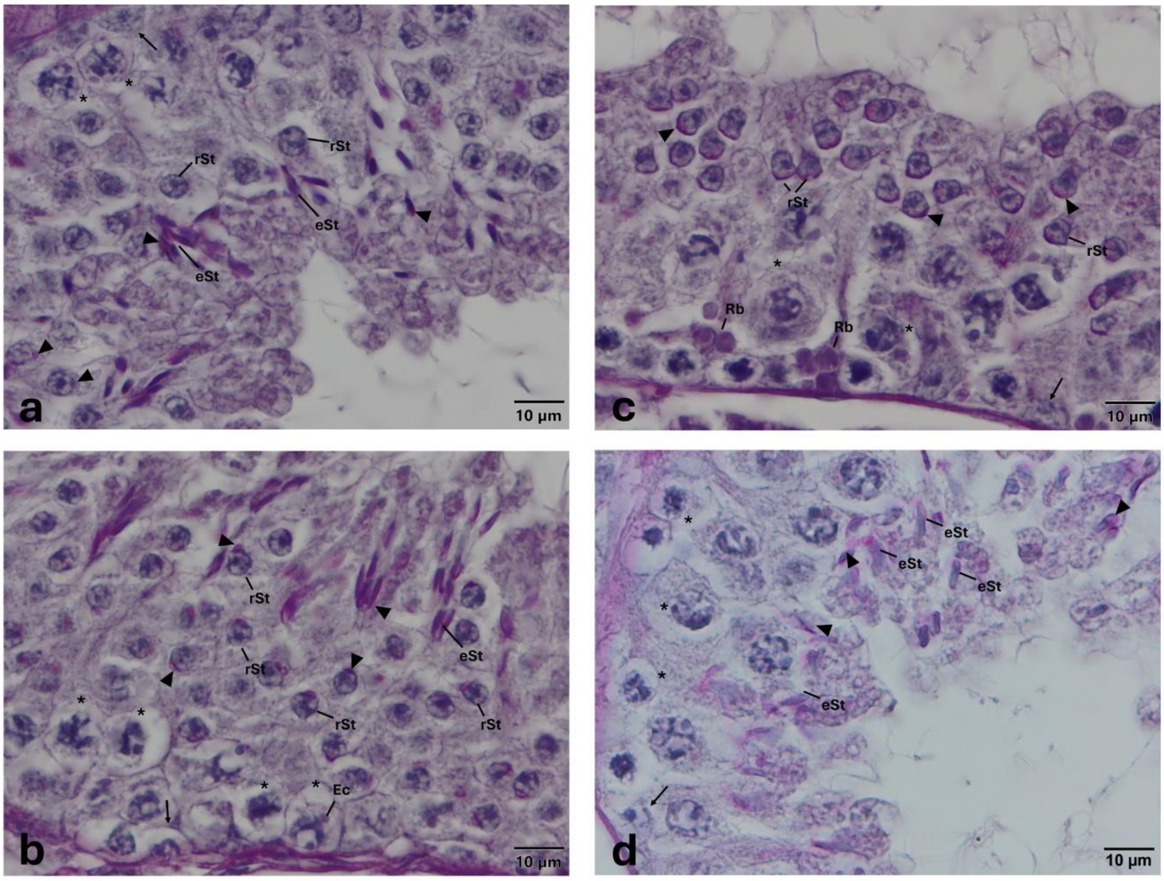

Supplementary Fig. 11 PAS staining on a sequential section for TLR-11 demonstrating the cycle of the seminiferous epithelium. The presence of round spermatids and/or elongated spermatids with PAS-positive acrosome reveals the cycle of the seminiferous epithelium (arrow heads). **a** Stage II-III; **b** Stage IV-V; **d** Stage VIII; **e** Stage X-XI. eSt: elongated spermatid; Ec: endosomal compartment; Rb: residual body; rSt: round spermatid; *: spermatocytes; arrows: spermatogonia.

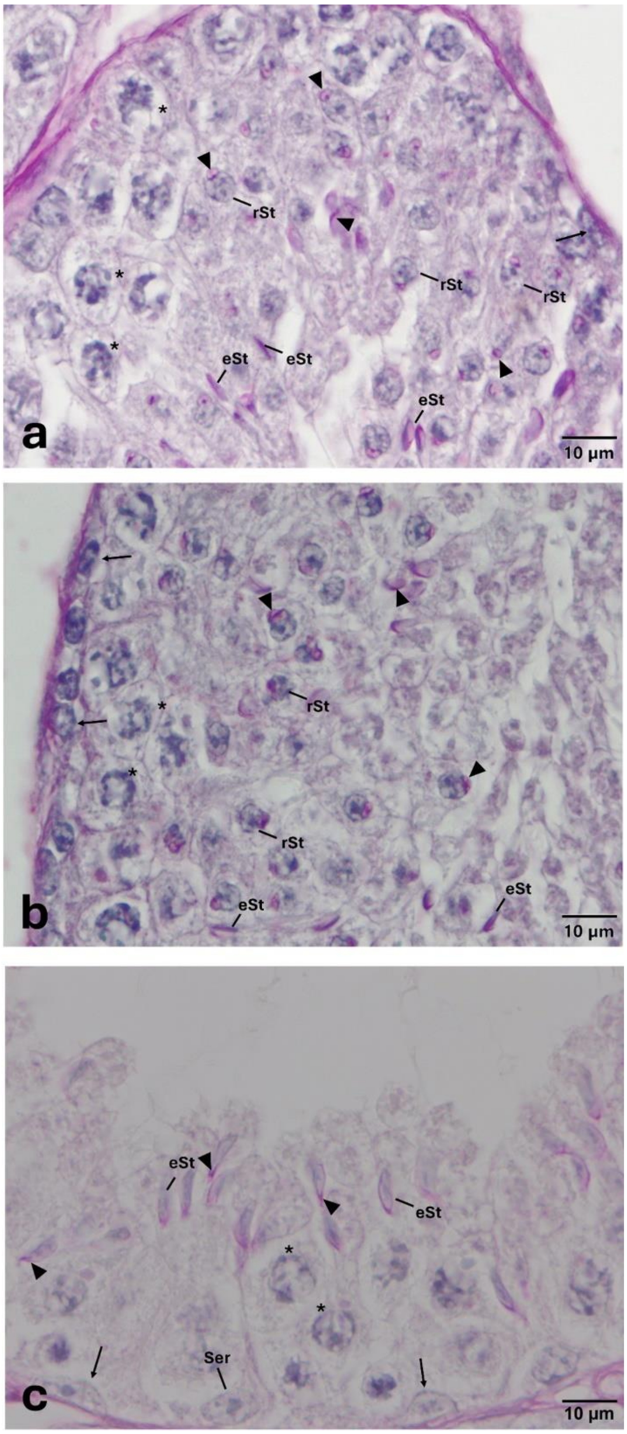

Supplementary Fig. 12 PAS staining on a sequential section for TLR-12 demonstrating the cycle of the seminiferous epithelium. The presence of round spermatids and/or elongated spermatids with PAS-positive acrosome reveals the cycle of the seminiferous epithelium (arrow heads). **a** Stage III-IV; **b** Stage VI; **c** Stage XI. eSt: elongated spermatid; rSt: round spermatid; Ser: Sertoli cell; *: spermatocytes; arrows: spermatogonia.

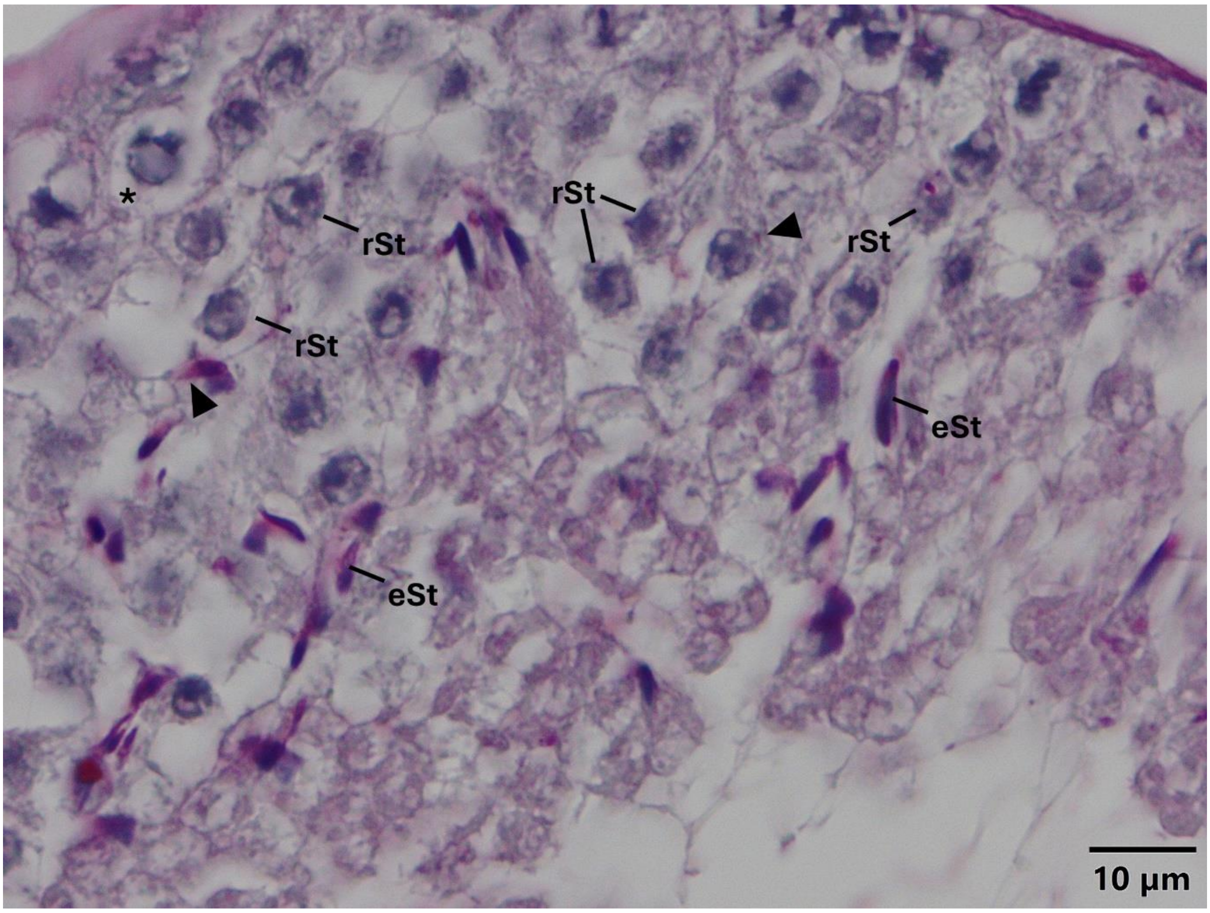

Supplementary Fig. 13 PAS staining on a sequential section for TLR-13 demonstrating the cycle of the seminiferous epithelium. The presence of round spermatids and/or elongated spermatids with PAS-positive acrosome reveals the cycle of the seminiferous epithelium (arrow heads) at stage I-II. eSt: elongated spermatid; rSt: round spermatid; *: spermatocytes.
